# Down‐regulation of the Sp1 transcription factor by an increase of microRNA-4497 in human placenta is associated with early recurrent miscarriage

**DOI:** 10.1186/s12958-021-00701-8

**Published:** 2021-02-12

**Authors:** Huaiyun Tang, Linqing Pan, Yun Xiong, Leilei Wang, Yugui Cui, Jiayin Liu, Lisha Tang

**Affiliations:** 1 Clinical Center of Reproductive Medicine, Lianyungang Maternal and Child Health Hospital, Kangda College of Nanjing Medical University, 222000 Lianyungang, Jiangsu China; 2 State Key Laboratory of Reproductive Medicine, Clinical Center of Reproductive Medicine, The First Affiliated Hospital of Nanjing Medical University, 210029 Nanjing, Jiangsu China

**Keywords:** Recurrent miscarriage, MicroRNA-4497, Sp1, Placenta, Chorionic Trophoblast

## Abstract

**Background:**

The pathophysiological mechanism of recurrent miscarriage (RM) is unclear. The goals of this study were to determine the role of microRNA-4497 overexpression in placental villus tissues in early RM; To identify the potential target mRNAs of miRNA-4497; And to investigate the microRNA-4497-mediated regulatory mechanisms in placental trophoblasts.

**Methods:**

Bioinformatics analysis was performed to identify the candidate target genes of miRNA-4497. The protein expression of Sp1 transcription factor (SP1), chemokine (C-X-C motif) receptor 5 (CXCR5) and bone morphogenetic protein 8a (BMP8A) were determined in the villus tissues of the RM and normal groups by Western blotting and immunohistochemistry. Cultured 293T cells were co-transfected with the miRNA-4497 agomir or luciferase reporter vectors containing the wild-type or mutant 3’-UTRs of the target mRNAs to verify the regulatory role of miRNA-4497.

**Results:**

Bioinformatics analysis suggested that SP1, CXCR5 and BMP8A mRNAs are potential targets of miRNA-4497. The expression of SP1, CXCR5 and BMP8A proteins in the chorionic villus tissues of RM placentas were significantly decreased compared to those in the normal controls. Moreover, SP1 protein levels were inversely correlated with the levels of miRNA-4497 in the placentas of RM patients and normal controls. The expression of SP1 mRNA and protein were down-regulated in HTR-8/SVneo cells after forced overexpression of the miRNA-4497 agomir. The results of the co-transfection assay showed that mutation of the miRNA-4497-binding sites in the 3’-untranslated region (3’-UTR) of SP1 led to a recovery of luciferase activity upon overexpression of miRNA-4497, suggesting that SP1 could be a direct target of miRNA-4497.

**Conclusions:**

An increased miRNA-4497 level in the placental villus tissues associated with recurrent miscarriage may down-regulate SP1 expression. The negative regulation of SP1 by miRNA-4497 may potentially contribute to the pathogenesis of recurrent miscarriage through promotion of trophoblast apoptosis. These findings provide novel information on the regulation of placental trophoblast apoptosis, and could be useful for the development of new therapeutic strategies for better management of recurrent miscarriage.

## Background

Miscarriage represents a major complication of pregnancy. Recurrent miscarriage (RM) is diagnosed when two or more consecutive miscarriages occur. Affecting 1–5 % of women in reproductive age worldwide, RM represents a serious health concern as well as an economic burden [1]. The risk factors for RM include structural uterine abnormalities, infection, autoimmune diseases, thrombophilia and metabolic imbalance [2]. Despite considerable research efforts in the past decades, the pathophysiological mechanism of RM remains largely unclear.

MicroRNAs (miRNAs) are small noncoding RNAs found in diverse organisms. miRNAs participate in the modulation of gene expression by binding to the 3’-untranslated regions (3’-UTRs) of the target mRNAs [3], resulting in changes in mRNA stability and/or suppression of protein translation. Subsequent changes in the protein expression could lead to pathophysiological alterations in the cell structure and function. Since the first identification of microRNA *lin-4* in *Caenorhabditis elegans*, the existence of a large number of miRNAs has been validated in humans. miRNAs regulate more than a third of human genes involved in a variety of biological processes, such as proliferation, differentiation, apoptosis, metabolism and cell migration and invasion [4]. In human, aberrant expression of miRNAs may contribute to RM and other reproductive disorders [5].

In our previous studies, a genome-wide screening of miRNAs identified several miRNA species with significantly altered steady-state levels in the chorionic villi of patients with early RM in comparison with normal pregnancy [6]. Particularly, miRNA-4497 was shown to be upregulated nearly 13-fold in the chorionic villi of RM patients. miRNA-4497 was initially identified in malignant human B cells [7] and is differentially expressed in THP-1 cells infected with mycobacterium tuberculosis [8]. Xiao et al. [9] reported that miRNA-4497 expression is down-regulated in the gastric stromal tumour tissues. In laryngeal squamous cell carcinoma, miRNA-4497 significantly promotes apoptosis of cancer cells, most likely through a negative modulation of the GBX2 (gastrulation brain homeobox 2) gene [10]. Moreover, several miRNAs, including miRNA-195, miRNA-376, miRNA-210 and miRNA-34a, have been reported to enhance or inhibit trophoblast migration and invasion, suggesting the potential roles of miRNAs in the regulation of placental function and development [11, 12]. Interestingly, human placenta may share some common biological features with malignancies due to a pattern of fast expansion and invasive growth. Human placenta in early pregnancy can be regarded as a physiological counterpart of malignant tumours, and placental trophoblasts may utilize a set of molecular pathways/mechanisms similar to those used by tumour cells [13].

We previously observed that the overexpression of miRNA-4497 significantly promotes trophoblast apoptosis [14]. However, the molecular target and pathologic mechanism of miRNA-4497 in the development of RM remained unknown. In the present study, software and databases were used to predict the potential targets of miRNA-4497. Multiple methods were applied to determine the correlations between the levels of miRNA-4497 and its target genes in the villus tissues of RM placentas. The findings of the present study may expand our understanding of the pathogenic mechanism of RM and provide potential therapeutic targets for better management of RM patients.

## Methods

### Ethics

This study was approved by the Ethics Committee of Lianyungang Maternal and Child Health Hospital, and informed consent was obtained from all women participating in the study.

### Human villus tissue, serum and cell lines

Paired samples of chorionic villus tissues were obtained from women undergoing dilation and curettage and classified into 2 groups: (1) the RM group, including pregnant women with RM, which was diagnosed for experincing two or more consecutive pregnancy losses within the first 12 weeks of gestation, and (2) the control group, including women with normal early pregnancy undergoing dilation and curettage. Patients with abnormal embryonic chromosomes, endocrine factors and anatomical factors and prethrombotic status were excluded from both groups to avoid interference by abnormal embryo growth. The chorionic villus tissues were thoroughly washed with sterile PBS to remove blood and mucus, and snap-frozen in liquid nitrogen for later isolation of RNA and proteins. Another set of tissue samples was fixed with 10 % formalin and embedded with paraffin for immunohistochemistry study.

Peripheral blood samples were collected from patients with RM or the control group upon their admission to the hospital. Serum was prepared by centrifugation at 3000 rpm for 15 min and stored at -80℃ for subsequent use.

BeWo and HTR-8/SVneo cells were obtained from the China Typical Culture Preservation Centre (Wuhan University, Wuhan, China). The BeWo cell line was derived from human choriocarcinoma, and have been widely used in the studies of trophoblast functions for more than 30 years. HTR-8/SVneo is an extravillous trophoblast cell line derived from the villus tissues of normal early pregnancy that has been extensively used for studying invasion of trophoblasts into the decidua.

### Cell culture and transfection

The cells were grown in F12K (BeWo) and RPMI 1640 media (HTR-8/SVneo) supplemented with 10 % foetal bovine serum (FBS), 100 µg/ml penicillin-streptomycin and 100 µg/ml penicillin (all Gibco, USA). Cell culture was maintained at 37℃ in an atmosphere containing 5 % CO_2_. miRNA-4497 agomir and negative control (NC) were purchased from RiboBio (Guangzhou, China). Cell cultures grown to 80 % confluence were transfected with the miRNA-4497 agomir/NC mixed with Lipofectamine 3000 (Invitrogen, USA) by following the manufacturer’s instructions. RNA was isolated 24 h after the transfection, and real-time PCR was performed to determine the levels of miRNA-4497 and twelve candidate target genes. The protein expression levels of SP1, CXCR5 and BMP8A in BeWo choriocarcinoma cells and HTR-8/SVneo trophoblasts were determined by Western blotting 48 h after the transfection.

### Quantitative real‐time PCR

Total RNA was extracted from the trophoblasts, placental villus tissues or serum using the Trizol reagent (Invitrogen, USA). The relative level of miRNA-4497 was measured using the One-Step SYBR® PrimeScript™ miRNA RT-PCR kit (Takara, Japan) according to the manufacturer’s protocol. cDNA was synthesized using the reverse transcription kit (Takara, Japan), and real-time PCR was performed using the SYBR green I mix (Takara, Japan). The thermocycling conditions were as follows: 95°C for 5 min, and 35 cycles at 95°C for 5 s and 60°C for 30 s. U6 small nuclear RNA and GAPDH mRNA were used as references for evaluation of miRNA-4497 and predicted target gene mRNA levels, respectively. Primer sequences for the detection of miRNA-4497 and its potential targets are shown in Table 1. All experiments were performed in triplicate, and the data were analysed by the 2^−ΔΔCt^ method.

**Table 1:** Primers used for RT-PCR validation

Gene symbol	Primer sequence (5’-3’)
**miRNA-4497**	F:GCGCGCTCCGGGACGG R:AGTGCAGGGTCCGAGTATT RT:GTCGTATCCAGTGCAGGGTCCGAGGTATTCGCACTGGATACGACGCCCAG
**U6**	F:AGAGAAGATTAGCATGGCCCCTG R:AGTGCAGGGTCCGAGGTATT RT:GTCGTATCCAGTGCAGGGTCCGAGGTATTCGCACTGGATACGACAAAATA
**GAPDH**	F:AATCCCATCACCATCTTCCA R:TGGACTCCACGACGTACTCA
**AGBL4**	F:CGCTTCCGAGTCTGGTTCAA R:CCATAGGGGCCATCCCATCT
**ESPN**	F: CAGAGTGCAGGACAAAGACAA R: GCAGCGTAGTGGATAGGCAG
**HTT**	F: AAACTTCTGGGCATCGCTATG R: GTTGAGGCATTCGTCAGCCA
**ISOC2**	F: ATCCTCTGTCCTGTTCCTGTG R: CTGAGACGATCTGTGGGAAGTA
**NRXN2**	F: CAGCACGAGGATGGATCGC R: GCCCACGTTAAAGATCACCCC
**RCC2**	F: AAGGAGCGCGTCAAACTTGAA R: GCTTGCTGTTTAGGCACTTCTT
**TAF6L**	F: CGGCGGTTTGTGGAGATCC R: CCTCTCTCAGACGATAGCACAC
**WASF2**	F: TAGTAACGAGGAACATCGAGCC R: AAGGGAGCTTACCCGAGAGG
**C20orf160**	F: ACCACGGCGGAGCAGGAC R: TAGGAGGCGGCGGCGATC
**SP1**	F: GGAAGGAGAAAACAGCCCAGAT R: GAGCCCCTTCCTTCACTGTCT
**BMP8A**	F:GTGTCAGGACAAGTCCCCTT R:TCCTGCAATGGCAGATAGGC
**CXCR5**	F:CACGTTGCACCTTCTCCCAA R:GGAATCCCGCCATGGTAG

### Western blotting analysis

Total cellular proteins were isolated from human tissues or transfected cells by using ice-cold radioimmunoprecipitation assay (RIPA) lysis buffer containing a protease inhibitor cocktail (Beyotime, cat# P0013C, China). Protein concentration was measured using a BCA protein assay kit (Beyotime, cat# P0012S, China) following the manufacturer instructions. Forty µg of proteins from each sample was resolved in 10 % SDS-polyacrylamide gels and electrophoretically transferred to PVDF membranes. After blocking with 5 % skim milk (Beyotime, cat# P0216-300 g, China) for 1 h, the membranes were incubated with SP1-specific antibody (dilution 1:2,000, product no. ab124804, Abcam, UK), CXCR5-specific antibody (dilution 1:5,000, product no. ab133706, Abcam, UK), BMP8A-specific antibody (dilution 1:1,000, product no. ab60290, Abcam, UK) or GAPDH-specific antibody (dilution 1:5,000, product no. 10494-1-AP, Proteintech, USA) with constant rotation at 4°C overnight. After three washes with TBST for 10 min each, the membranes were incubated with HRP-conjugated anti-rabbit IgG antibodies (1:2,000, product no. ab187910, Abcam, UK) at room temperature for 1 h. After washing, the signals were developed using the ECL (San Diego, USA) system. The densitometry analysis was performed using Quantity One software (Bio-Rad, USA).

### Immunohistochemistry

After fixing with 4 % formalin and paraffin-embedding, villus tissues were sectioned (5 µm). The sections were incubated with primary antibodies to SP1 (1:200, Abcam, product no. ab124804, UK), CXCR5 (1:200, Abcam, ab133706, UK) or BMP8A (1:200, Abcam, product no. ab60290, UK) after blocking with 5 % (*w/v*) bovine serum albumin (BSA). The sections were washed 3 times with PBS and subsequently incubated with the horseradish peroxidase (HRP)-conjugated secondary antibody (CWBIO, CW2069S, China), at 37 ℃ for 1 h. The sections were stained with diaminobenzidine (DAB) (CWBIO, CW2069S, China) and mounted for bright-field microscopy (Bremen, Germany). Immunohistochemical experiments were repeated at least 3 times for each villus sample.

### Dual luciferase reporter assay

The wild-type (SP1-WT, CXCR5-WT and BMP8A-WT) and mutated (SP1-MUT, CXCR5-MUT and BMP8A-MUT) 3’-UTRs were produced by PCR using genomic DNA of 293T cells as templates and primers containing wild-type or mutated sequences (primer sequences are shown in Table 2). The resulting DNA fragments were subcloned into the pmiR-RB-REPORTTM dual luciferase reporter vector (Ribobio, China). For transfection, the cells were seeded onto 96-well plates at 40–50 % confluence. After overnight culture, the cells were co-transfected with the reporter plasmids containing wild-type or mutated 3’-UTR of the target genes, and with miRNA-4497 agomir or the negative control using Lipofectamine 3000 (Invitrogen, cat# L3000015, USA). The luminescence signals corresponding to the activities of Renilla and firefly luciferases were measured. The firefly luciferase activity was used as an internal control for standardization of the transfection efficiency. Relative changes in the Renilla luciferase activity were calculated and compared.

**Table 2:** Primers used for luciferase reporter assay

Vectors	Primer sequence (5’-3’)
**SP1-3UTR**	F:GGC GCT CGA GAG GGG CCC TTT GCA TAG CTC TCC TT R: AAT GCG GCC GCG GGA AAG CAC ACA TCC CAG CAG AGG TA
**SP1-MUT-3UTR**	F: TCT CCA ACG GGC CTA AAC ATT GCT TTT GAA AAC TGC R: CAA TGT TTA GGC CCG TTG GAG AAG TGC CAT ATC
**CXCR5-3UTR**	F: GGC GCT CGA GGA TGG GAG GTT GTG GGC ATT GAT GG R: AAT GCG GCC GCG GCT TGT TCC GGG GGT CTC TGT GCT
**CXCR5-MUT-3UTR**	F: GGC TTG TAG GCC CGG TCT CTG TGC TGC CTG TA R: AAT GCG GCC GCG GCT TGT AGG CCC GGT CTC TGT G
**BMP8A-3UTR**	F: GGC GCT CGA GGT CCT GTG TCT TGG GTC TGT GAG TC R: AAT GCG GCC GCG GTC ATG GGG GCA GAT GGG TTC TCT
**BMP8A-MUT-3UTR**	F: TCC ACT GTA GGG CCT AAC CGC TGT TCT CCT TGG A R: CAG CGG TTA GGC CCT ACA GTG GAG GCC GAG ACA A

### Apoptosis assay

HTR-8/SVneo trophoblast cells were plated in 6-well plates and transfected with negative control siRNA or specific siRNA for 24 h. Cells were digested with EDTA-free trypsin, washed twice with cold PBS, and centrifuged at 1000 rpm for 5 min. The cells were re-suspended in 500 µl binding buffer and stained with 5 µl of Annexin V-FITC (AV) and 5 µl propidiumiodide (PI) from the Annexin V-FITC/PI staining kit (KeyGene BioTech). The status of cell apoptosis was analyzed by flow cytometry (BD ACCURI C6).

### Statistical analysis

All data were analysed using SPSS 16.0 software (SPSS, Inc., Chicago, IL, USA). Statistical significance was determined by one-way ANOVA (PRISM software version 3.03; GraphPad) followed by Scheffe’s post hoc test. *P* < 0.05 was considered to indicate statistical significance.

## Results

### miRNA-4497 was highly expressed in villus and serum of RM patients

**Fig. 1:**
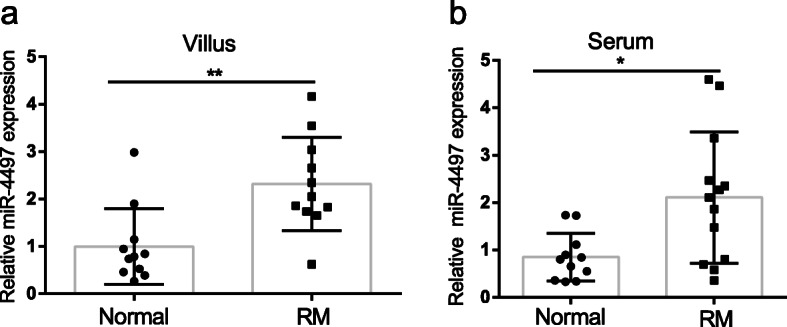
Increased miRNA-4497 levels in placental villus tissues and serum of RM patients. **a** miRNA-4497 levels in villus tissues from RM (*n* = 11) patients and normal control women (*n* = 11). **b** miRNA-4497 levels in the serum of RM (*n* = 11) patients and normal control women (*n* = 13). *, *P*<0.05; **, *P*<0.01

In order to explore the possible role of miRNA-4497, we firstly detected the expression of miRNA-4497 in chorionic villi of early recurrent miscarriage and normal control women. Real-time PCR confirmed the increased expression of mature miRNA-4497 in villus tissues of RM patients compared with healthy control women (Fig. 1a). The expression of miRNA-4497 in serum of RM and normal control group was detected. Consistent with the changes in villus tissues, the miRNA-4497 levels were significantly increased in the serum samples of RM patients (Fig. 1b). These observations suggested that miRNA-4497 could be a potential biomarker in the diagnosis of RM.

### Identification of BMP8A, SP1 and CXCR5 as the candidate target genes of miRNA-4497

**Fig. 2:**
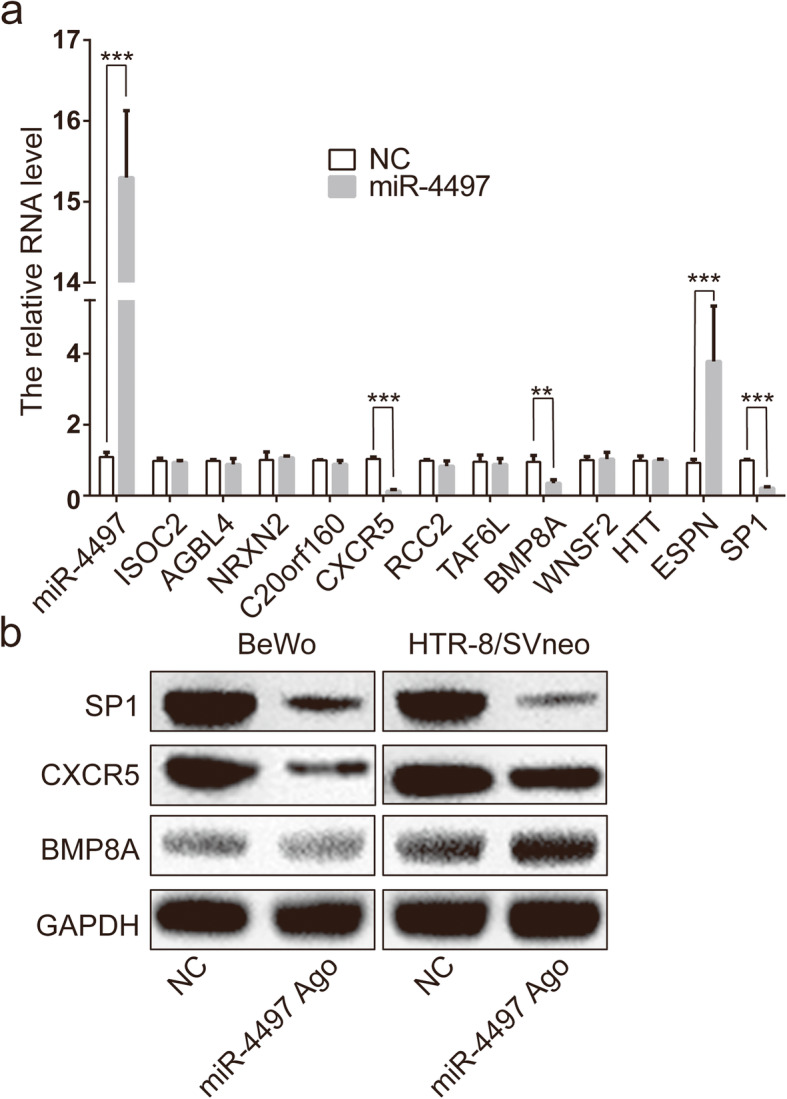
Expression levels of predicted target genes in the trophoblasts after the transfection with miRNA-4497 agomir. **a** Levels of miRNA-4497 and mRNAs of 12 predicted target genes measured by real-time PCR after the transfection of HTR-8/SVneo cells with miRNA-4497 agomir or scrambled sequences used as a negative control (NC). Note the significant reduction in SP1, CXCR5 and BMP8A mRNA levels after overexpression of miRNA-4497. **b** The protein expression levels of SP1, CXCR5 and BMP8A determined by Western blotting after transfection of BeWo and HTR-8/SVneo trophoblast cell lines with miRNA-4497 agomir or negative control (NC). Note the significant decrease in the protein levels of SP1 and CXCR5 after overexpression of miRNA-4497. *, *P*<0.05; **, *P*<0.01; ***, *P*<0.001

To determine the regulatory mechanisms of miRNA-4497, the potential targets of miRNA-4497 were predicted using bioinformatics tools, miRanda (www.microrna.org/microrna/), microCosm (www.ebi.ac.uk/enright-srv/microcosm/htdocs/targets/v5/) and Targetscan (www.targetscan.org/). Numerous target genes of miRNA-4497 were identified, and twelve genes detected by all three miRNA databases as potential targets were selected for validation [6]. The relative levels of these twelve candidate target genes was determined with the use of real-time PCR after the transfection of BeWo and HTR-8/SVneo cells with miRNA-4497 agomir. As expected, the results showed that the miRNA-4497 level was significantly increased after cell transfection (Fig. 2a). The endogenous mRNA levels of twelve target genes including those of *SP1, CXCR5* and *BMP8A* were significantly decreased compared with those in the negative control, and the ESPN mRNA level was significantly increased (Fig. 2a). Moreover, the results of the Western blotting assay showed that overexpression of miRNA-4497 lead to a significant decrease in the protein levels of SP1 and CXCR5 in both cell lines (Fig. 2b).

### Correlations between the levels of miRNA-4497 and the target genes in chorionic villus tissues

**Fig. 3:**
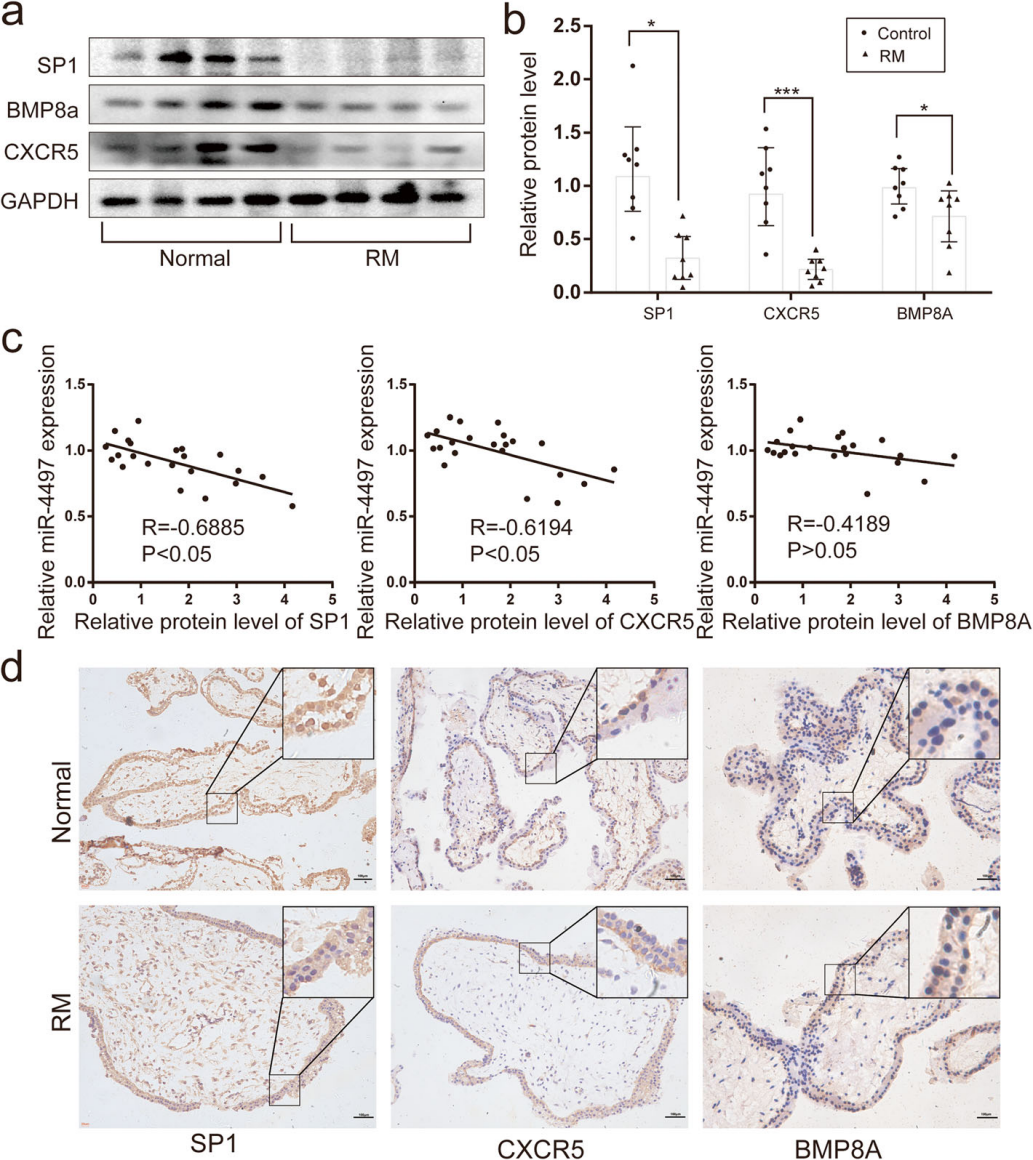
SP1 levels are inversely correlated with miRNA-4497 levels in the placental villus tissues. **a** and **b** Decreases of the SP1, CXCR5 and BMP8A protein levels in RM (*n* = 8) compared with those in normal chorionic villus tissues (*n* = 8). **c** The protein levels of SP1 and CXCR5, but not BMP8A, are inversely correlated with the miRNA-4497 levels in chorionic villus tissues from RM and normal control patients. **d** The results of immunohistochemical analysis of SP1 expression in the villus tissues of RM and normal controls. A significantly low SP1 level was detected in the villus tissues of RM. There were no significant change in the expression of CXCR5 and BMP8A. Villus tissues were counterstained with haematoxylin. SP1, CXCR5 and BMP8A proteins were stained in brown colour. *, *P*<0.01, ***, *P*<0.001

To determine the regulatory role of miRNA-4497 *in vivo*, the protein expression of SP1, CXCR5 and BMP8A was assayed in the RM and normal chorionic villus tissues by Western blottting, and the correlation analysis was performed. The levels of the three proteins were significantly decreased (*p < 0.05) in the chorionic villus tissues of RM placentas (Fig. 3a and b). Immunohistochemistry was performed to confirm these results and to determine protein localization. SP1 protein was readily detected in the nuclei of trophoblasts in the villus tissues from normal pregnancy (Fig. 3d). The SP1 protein expression was lower in the trophoblasts in the RM samples compared with that in the normal group (Fig. 3d). Importantly, the Spearman correlation analysis showed that SP1 and CXCR5 protein levels were inversely correlated with the miRNA-4497 levels (Fig. 3c), suggesting that SP1 may be involved in RM pathogenesis. Semi-quantitative analysis of immunohistochemistry results (Fig. 3d) showed that while SP1 protein levels were significantly decreased in RM villus trophoblasts compared with control samples (The percentage of SP1 positive stain cell is 12.2 ± 0.8 % and 18.3 ± 1.1 %, respectively, *P*<0.05), the CXCR5 and BMP8A protein expression levels showed a trend of reduction in the RM placental trophoblasts, but the difference did not reach a statistical significance (The percentage of CXCR5 positive cells is 7.3 ± 0.4 % and 8.5 ± 0.4 %, respectively,* P* > 0.05; The percentage of BMP8A positive cells is 8.0 ± 0.5 % and 8.6 ± 0.3, respectively, *P* > 0.05), suggesting that CXCR5 may be less likely related to RM pathogenesis than SP1does. For this reason, we elected to concentrate on SP1 for further investigation.

The BMP8A mRNA level was decreased after the transfection of the cells with miRNA-4497, but the protein level was not significantly altered after the transfection. This discrepancy between mRNA and protein levels could be caused by an unknown regulation of BMP8A protein that may offset the decrease of BMP8A mRNA, e.g., a stabilization of BMP8A protein after transfection of the cells with miRNA-4497. Alternatively, if BMP8A protein has an extraordinarily long turnover time, examination of protein levels at 48 hours post-transfection may not be sufficient to observe a change. The observation that BMP8A protein level was not altered in the trophoblasts of the RM villus tissues seems to support the former possibility. Whatever the reason may be, a lack of changes in BMP8A protein in the RM villus tissues seem to suggest that BMP8A protein may be unlikely related to the pathogenesis of RM.

### SP1 is the direct target of miRNA-4497

**Fig. 4:**
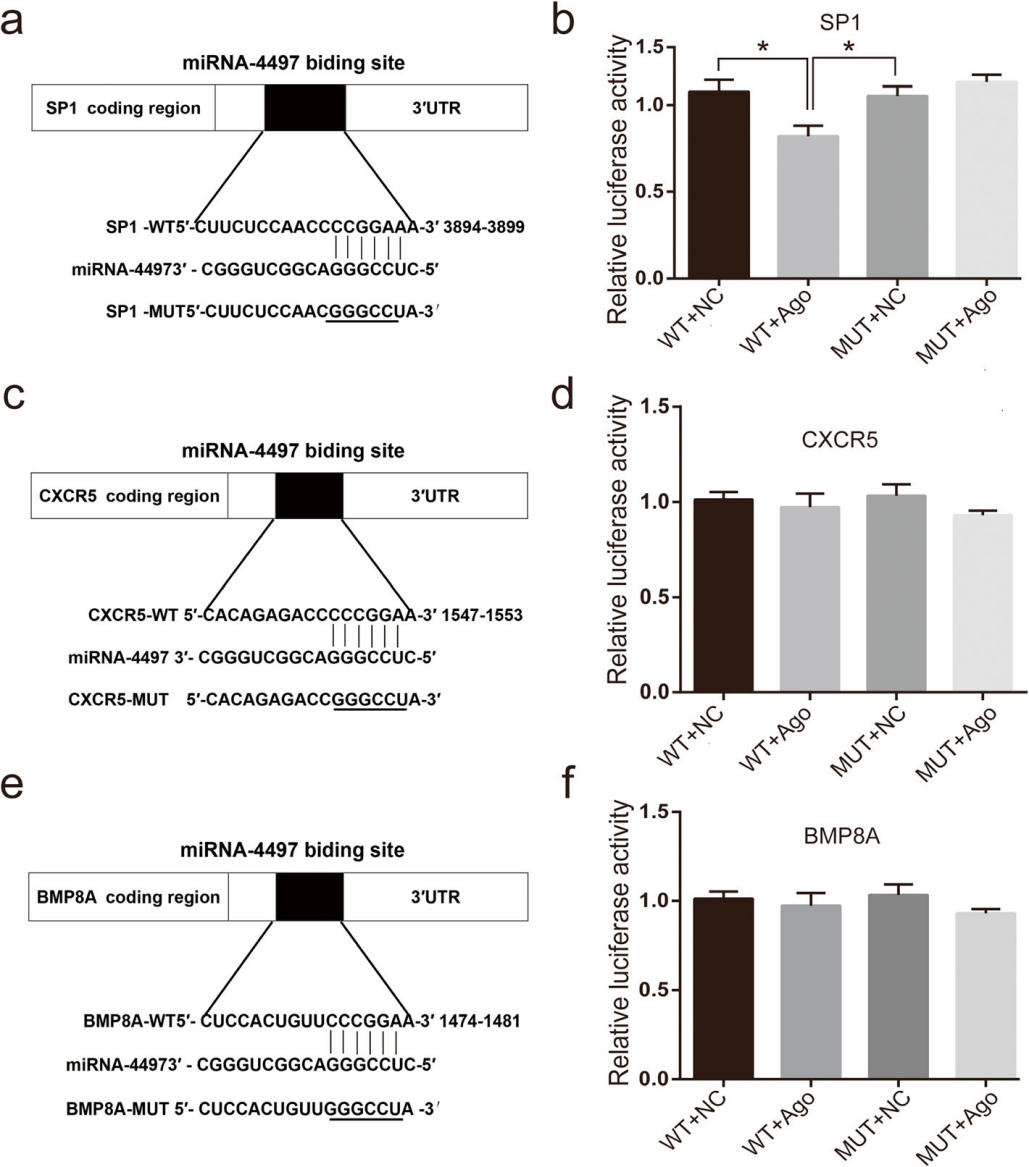
SP1 mRNA is the direct target of miRNA-4497. Cultured 293T cells were co-transfected with the reporter vectors containing wild-type (WT) or mutated (MUT) 3’-UTRs of the target genes with miRNA-4497 agomir (Ago) or scrambled sequence used as a negative control (NC) using Lipofectamine 3000. The left panels (**a**, **c**, **e**) illustrate the data of the assay in the cells co-transfected with miRNA-4497 sequences and 3’-UTR sequences of SP1, CXCR5 and BMP8A. Mutations of the potential miRNA-4497-binding sites are underlined. The right panels (**b**, **d**, **f**) present relative changes in the luciferase activity after co-transfection of HTR-8/SVneo trophoblasts. **b** Overexpression of miRNA-4497 induced a reduction in the SP1 3’-UTR reporter activity (WT + Ago) compared to that in the control (WT + NC). Mutation of the binding site in SP1 3’-UTR resulted in the recovery of reporter activity. No significant changes in the luciferase activity of the reporters containing the 3’-UTR of CXCR5 and BMP8A were detected after co-transfection of HTR-8/SVneo trophoblasts with miRNA-4497 agomir. Additionally, mutation of the miRNA-4497-binding sites did not influence the reporter activity in the co-transfection assay. *, *P*<0.01

The bioinformatics analysis suggested that SP1, CXCR5 and BMP8A are the potential targets of miRNA-4497 and identified the presence of the putative binding sites of miRNA-4497 in the 3′-UTRs of the target mRNAs (Fig. 4a, c and e). To verify the significance of these binding sites, the wild type as well as mutated sequences complementary to the miRNA-4497 binding sites in the SP1, CXCR5 and BMP8A 3′-UTRs were subcloned into the luciferase reporter vector (Fig. 4a, c and e), and the effects were tested in a co-transfection assay in 293T cells. The data indicated that the luciferase activity of the vector containing the wild-type SP1 3′-UTR was decreased by co-transfection with miRNA-4497 agomir compared with that in the control (Fig. 4b). Moreover, the luciferase activity was recovered if the binding sites in the SP1 3′-UTR was mutated (Fig. 4b), suggesting that miRNA-4497 may directly target SP1 mRNA. Additionally, the luciferase activity was not changed if miRNA-4497 agomir was co-transfected with the reporter vectors containing mutated 3′-UTRs of CXCR5 or BMP8A or if the vector expressing scrambled RNA sequences was co-transfected with the reporter vector containing wild-type 3’-UTRs of CXCR5 or BMP8A (Fig. 4d and f). This result suggested that the decreased mRNA levels of CXCR5 and BMP8A following overexpression of miRNA-4497 (Fig. 2) may not be a direct effect by the binding of miRNA-4497 to the studies sites in these two 3′-UTRs. The regulatory mechanisms of miRNA are known to be complicated and sometimes illusive. miRNA-4497 may either exert its effect through binding to alternative sites or indirectly through an action involving the other factor(s), which ultimately leads to the reduction of CXCR5 mRNA. Overall, these results indicated that SP1, but not CXCR5 and BMP8A, may be the direct target of miRNA-4497. These observations are mostly consistent with the results of the correlation studies shown in Fig. 3.

### Knock down of SP1 produced similar apoptotic effects to miRNA-4497 overexpression in HTR-8/SVneo trophoblasts

**Fig. 5:**
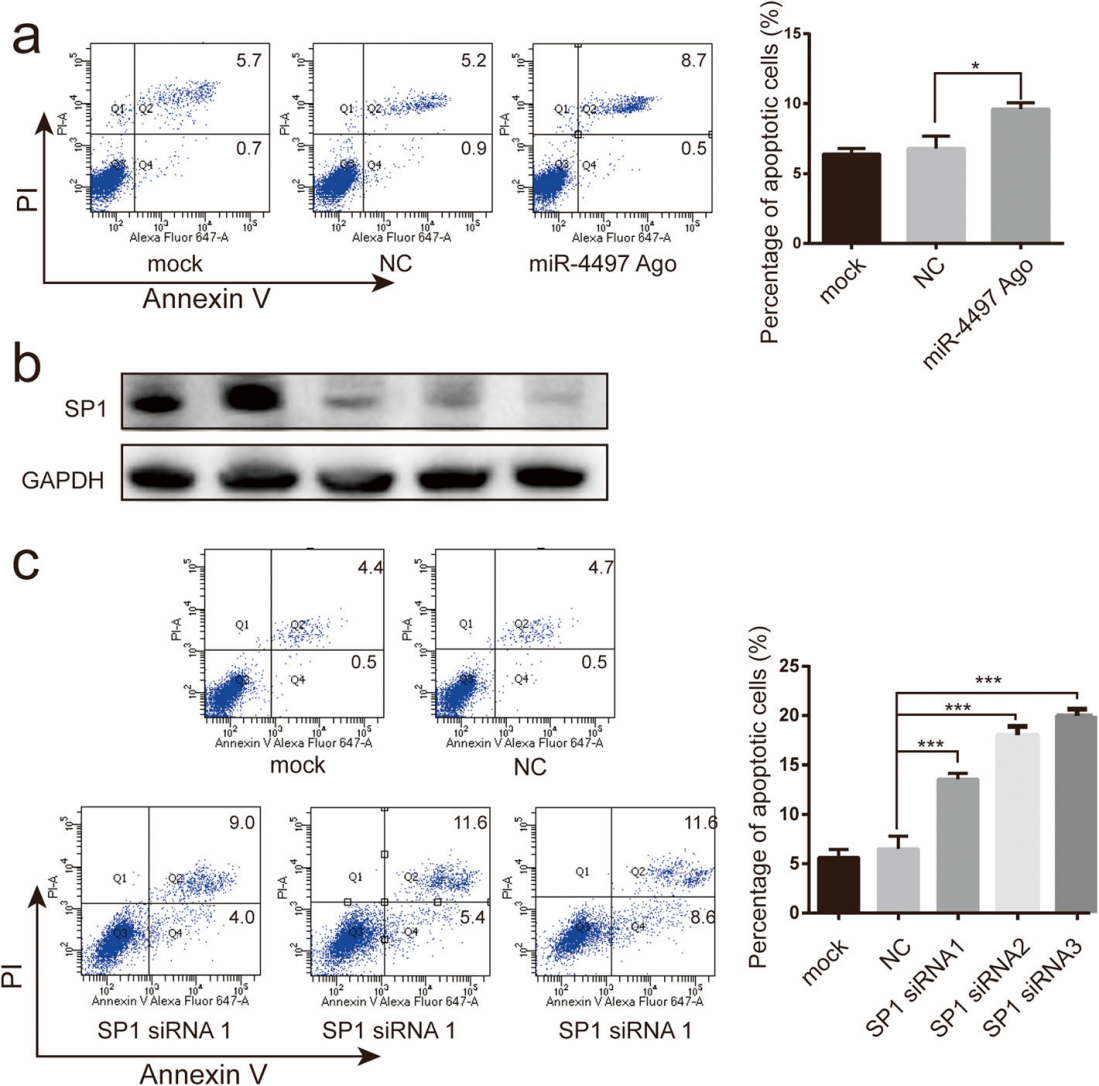
miRNA-4497 induced trophoblast apoptosis by targeting SP1. (a) Effect of miRNA-4497 on trophoblast cell apoptosis was detected by apoptosis assay. miRNA-4497 overexpression significantly induced trophoblasts apoptosis. (b) Western blotting was used to detect the effect of SP1-siRNA on the expression of SP1. Transfection of SP1-siRNA into trophoblast significantly reduced the SP1 protein expression. (c) Effect of SP1 on trophoblasts apoptosis was detected by apoptosis assay. SP1 under-expression led to an increased percentage of apoptotic cells in HTR-8/SVneo culture. *, *P*<0.05; ***, *P*<0.001

To explore the potential effect of miRNA-4497 on trophoblasts, we examined the cell apoptosis following overexpression of miRNA-4497. Compared with the negative control, miRNA-4497 agomir treatment significantly increased the apoptosis of HTR-8/SVneo cells (Fig. 5a). In order to clarify whether the increased apoptosis mediated by miRNA-4497 could be dependent on its regulation on SP1, we also determined the effect of SP1 manipulation in the context of cell apoptosis. After 48 h transfection with SP1-siRNA, the expression of SP1 protein was significantly decreased (Fig. 5b). As showed in Fig. 5c, when SP1 expression was down-regulated, the percentage of apoptotic cells was obviously increased (Fig. 5c). These results suggested that miRNA-4497 induced trophoblast apoptosis by targeting SP1.

## Discussion

Pregnancy is an elaborate and complex process involving the central events of placental implantation and embryo development. Dysregulation of placental functions is associated with various complications of early pregnancy, including RM [15]. Trophoblasts constitute a cellular component of the most importance for placental implantation and maturation. Human trophoblasts are tightly regulated by many transcriptional factors, extracellular matrix and certain adhesion molecules. In recent years, numerous non-coding miRNAs have been detected in human placentas, and some miRNAs have been shown to play a role(s) in the regulation of the trophoblast functions [16]. For example, it was reported that miRNA-431 is able to inhibit the migration and invasion of the trophoblasts by targeting ZEB1 [17]. miRNA-520 may promote DNA damage-induced trophoblast apoptosis in RM [18]. miRNA-let-7d suppresses the proliferation and invasion of trophoblasts, which may contribute to the pathogenesis of preeclampsia [19]. Our previous studies demonstrated that miRNA-4497 was expressed at a high level in placentas associated with RM, and its overexpression promotes apoptosis of chorionic trophoblasts [14]. In this study, we also observed a higher level of circulating miRNA-4497 in patients with RM than that in the control group with normal pregnancy. The data of the present study demonstrate a significant reduction in the levels of three candidate target mRNAs of miRNA-4497 in the villus tissues of RM placentas compared with normal placentas. Additional assays of the protein levels and luciferase reporter activity indicated that SP1 mRNA can be a direct target of miRNA-4497 in placental trophoblasts.

Down-regulation of miRNA-4497 has been detected in several human tumours including pancreatic cancers, gastric stromal tumours and laryngeal squamous cell carcinomas [9, 10, 20]. In laryngeal squamous cell carcinoma (LSCC), miRNA-4497 was shown to inhibit the proliferation and induce apoptosis of the LSCC cells, a similar effect to what we previously observed in placental trophoblasts. Moreover, the overexpression of miRNA-4497 appeared to be associated with the decreased mRNA and protein levels of Bcl2 and GBX2 in LSCC cells [10]. In the present study, bioinformatics analysis identified twelve target genes of miRNA-4497, and real-time PCR assay validated that the high miRNA-4497 levels inversely correlated low mRNA levels of SP1, CXCR5 and BMP8A in the villus tissues of RM placentas. Overexpression of miRNA-4497 specifically inhibited the luciferase activity of the reporter vectors containing the 3′-UTR of SP1. SP1 is one of the 26 members of the SP/KLF transcription factor family, which recognizes a GC-rich site in the promoters of many genes [21]. Previous studies demonstrated that overexpression of SP1 promoted cell proliferation and tumour growth. Luo et al. [22] reported that silencing SP1 expression results in a reduced invasiveness of glioma cells. In gastric cancer (GC), SP1 was found to be associated with oncogenesis and to promote cancer cell growth and metastasis [23]. In placenta, dysfunction of trophoblasts may cause reproductive disorders such as preeclampsia, foetal growth restriction, abnormal implantation and RM [12]. It was reported that SP1 regulates the expression of 11-hydroxysteroid dehydrogenase type 2 (11β-HSD2) in human placental trophoblasts [24]. Li et al. [25] showed that SP1 enhances trophoblast migration and invasion. Overall, the findings from the present as well as previous studies suggest that overexpression of miRNA-4497 in RM placenta may down-regulate the SP1 expression through destabilization of SP1 mRNA, and a decrease in the SP1 expression could negatively impact trophoblast functions.

CXCR5, another predicted target gene of miRNA-4497, was implicated in the regulation of cell proliferation, invasion and migration in breast cancer [26], hepatocellular carcinoma [27], myeloma [28] and other malignancies [29]. Our data indicated that CXCR5 was down-regulated in the villus tissues of RM placentas. Overexpression of miRNA-4497 decreased the expression of CXCR5 at the mRNA and protein levels in trophoblasts. However, unlike SP1, the luciferase activity from the vector containing the CXCR5 3′-UTR was not influenced by miRNA-4497 overexpression. Moreover, the mutations of miRNA-4497 or the CXCR5 3′-UTR did not change the reporter activity, suggesting that CXCR5 is unlikely a direct target of miRNA-4497. Thus, CXCR5 could be down-regulated by miRNA-4497 through an alternative, indirect pathway. Further investigations are required to reveal the underlying mechanism.

Due to the limited amount of villous tissues, we were unable to perform more sophisticated experiments to verify the physical interactions between miRNA-4497 and the 3’-UTR of SP1 mRNA. Another drawback of the current study is the lack of data on additional effects of miRNA-4497 e.g., on cell migration and invasion in the primary culture of trophoblasts. Moreover, applying the relatively simple criteria for the diagnosis of RM, and the use of few superficial exclusion conditions for the RM group, we could not avoid cases that were simply due to overlapping miscarriages caused by diversified reasons. This misdiagnosis would inevitably weaken the power of our observations. Further examination of miRNA-4497 and SP1 expression levels in miscarriage cases may help to verify how specific the observed changes are for RM. In addition, *in vivo* manipulation of miRNA-4497 using animal models is required to verify the physio-pathological role(s) of miRNA-4497 for the regulation of placental structure and function.

## Conclusive remarks

Findings from the present study indicate that an increased level of miRNA-4497 is present in RM placentas, and a forced overexpression of miRNA-4497 may down-regulate the apoptosis of placental trophoblasts by targeting SP1, which may be related to the development of RM. The new data enhances our understanding of the pathogenesis of RM, and could be useful for the development of novel strategies for early diagnosis and treatment of recurrent miscarriages.

## Data Availability

All data generated or analysed during this study are included in this published article.

## Abbreviations

RM: Recurrent miscarriage
SP1: Sp1 transcription factor
CXCR5: Chemokine (C-X-C motif) receptor 5
BMP8A: Bone morphogenetic protein 8a
ESPN: Espin; 3'-UTR: 3'-untranslated region
GBX2: Gastrulation brain homeobox 2
LSCC: Laryngeal squamous cell carcinoma
GC: Gastric cancer
11β-HSD2: 11-hydroxysteroid dehydrogenase type 2
